# The influence of actors on the content and execution of a bereavement programme: a Bourdieu-inspired ethnographical field study in Sweden

**DOI:** 10.3389/fpubh.2024.1395682

**Published:** 2024-05-23

**Authors:** Hakima Karidar, Pia Lundqvist, Stinne Glasdam

**Affiliations:** ^1^Integrative Health Research, Department of Health Sciences, Faculty of Medicine, Lund University, Lund, Sweden; ^2^Palliative and Advanced Homecare (ASIH) Lund, Lund, Sweden

**Keywords:** bereavement support programme, Bourdieu, children, ethnographic field study, parental death, volunteers

## Abstract

**Introduction:**

The death of a parent can have profound negative impacts on children, and a lack of adequate support can exacerbate negative life experiences.

**Aim:**

To explore the influences of various actors on the content and execution of a bereavement programme within a Swedish context, considering relational and contextual perspectives.

**Methods:**

An ethnographic field study involving six children, their parents, and eight volunteers. A theory-inspired thematic analysis was conducted, methodically inspired by Braun and Clarke, theoretically inspired by Bourdieu’s concepts of position, power, and capital.

**Results:**

Confidentiality obligation was an essential element in the programme, however, the premisses varied depending on actors’ positions. Volunteers and researchers had different outlets to express their experiences in the program. The programme offered the children an exclusive space for talking about and sharing experiences and feelings. Simultaneously, the programme restricted the children by not allowing them to share their experiences and feelings outside the physical space. The physical settings shaped the different conditions for interactions among the actors. The sessions adopted loss-oriented approaches, where communication between volunteers and children was guided by the volunteers. However, children created strategies for additional, voiceless communication with their peers or themselves. During breaks and mingles, shared interests or spaces connected children (and adults) more than their common experience of parental bereavement.

**Conclusion:**

The participants in the programme were significantly influenced by the structural framework of the programme, and their positions within the programme provided them with different conditions of possibility for (inter)acting. Children’s daily activities and interests were both ways to cope with parental bereavement and connect them to other people.

## Introduction

The death of a parent during childhood can have detrimental consequences in a child’s life, and a lack of support can potentially increase some children’s vulnerability to negative life experiences (1). Previous research shows that parental death is associated with an increased risk of depression and psychological disorders (2, 3), subsequent poor school performance, decreased socio-economic conditions (4, 5), criminality (6), self-inflicted injuries (7), mortality in childhood, and suicide later in life (8, 9). However, research also shows that distressing experiences of losing a parent during childhood’s formative years can pave the way to personal development, and that children have remarkable resilience and an ability to navigate through the complexities of life (10–12). Lund (13), argues that grief should be viewed as a natural emotion of loss. In many ways, experiences of loss have significance for people’s self-understanding, self-relation, and relationships with others in society (13).

The management of death and grief is influenced by culturally mediated experiences and social norms (13). In western societies, bereaved individuals can find themselves isolated and struggle to navigate their new life situation due to a lack of communication with people close to them, which may impact their well-being and ability to cope with their loss and the bereavement they are experiencing (14–16). Other studies show that some children perceive a taboo around death, due to adults perceiving death and bereavement as difficult subjects to talk about (17, 18). According to Wray et al. (19), some remaining parents avoid open communication with their children regarding loss and grief in an attempt to protect them. However, children are often more aware of death than is expected by adults. Thus, some of these children suppress their emotions, withdraw from social activities, and the support offered by adults does not always align with the children’s needs (17, 19). Some children face difficulties finding legitimate ways to cope with parental bereavement and the associated grief (20).

Research highlights childhood bereavement as a public health issue that requires engagement not only from professionals but also from communities to gain a deeper understanding and provide appropriate support for bereaved children (19). Nevertheless, professionals in healthcare settings and schools frequently express feelings of emotional strain, inadequacy in their abilities and training, and various other challenges such as lack of time and poor collaboration with other professionals when it comes to supporting children in their grief. Often, professionals perceive such tasks as falling outside the scope of their expertise, leading to them referring grieving children to other professionals and/or bereavement support programmes (21–24).

In western societies, individuals’ experiences, including the handling of bereavement and grief, have become both a personal and professional matter (16, 25). It is a personal matter in the sense that experiences of disease and bereavement are handled by individuals, maybe shared with some close family members (16). It is a professional matter as grief reactions are often regarded as potential pathological conditions, which may require psychological and/or pharmaceutical interventions to support the individual’s recovery. The primary objective of professional interventions is to facilitate people in their recovery from bereavement as quickly as possible, so they can resume their lives as if nothing significant has happened to them (13, 26). At the same time, professionals claim that the guardian parents are responsible for providing support both during and after a parent’s death (22, 27). Some parents seek support for their children from professionals such as teachers, social workers, and professional led support programmes, either through their own initiatives or at the request of others (16, 23). This is often because the capacity of the remaining parents to address their child’s needs may be constrained since they are also experiencing a loss, namely of their co-parent (28). However, it is important to consider that some children do not prefer professional support, and some of the parents ignore children’s wishes. This can force the children to accept parents’ decisions such as participating in support programmes (23, 29).

During the last 6 decades, the professionalisation of bereavement and grief management has manifested itself in the development of various individual and group support programmes, aiming to support self-help (16). During the 1980s, different psychological programmes were developed to support bereaved children and their families [see, for example, (30–36)]. Evaluations of different programmes show different positive outcomes, significantly impacting children’s behaviour, sleeping patterns, anxiety, and depressive symptoms (30, 34), and positive impacts on children’s self-esteem and improved communications skills between children and parents (31, 36). All in all, these cognitive psychological-inspired programmes have similar structures, consisting of different sessions with themes for reflection, including problem identification and the articulation of children’s feelings and emotions. Moreover, during the last decade, new concepts of support programmes have come about, considering children’s own perspectives, and involving them in the development of future programmes. Consequently, researchers have argued for the value of democratic processes and cooperation with children, where children are regarded as active co-actors in the designing, planning, and implementation of bereavement programmes (37–39). Overall, the primary focus and outcomes of intervention programmes are often presented based on existing theoretical approaches, with the emphasis being placed on uncovering ‘what’ works. Few studies have focused on the ‘why’ or ‘how’ behind what works (40, 41). Basically, all different kinds of group interventions are performed in social contexts. However, research often lacks consideration of the importance of contextual influence and the relational dimension, understood as how different actors impact the content and implementation when evaluating the outcomes of any intervention programme (40). To the best of our knowledge, previous research has not explored how and in which ways different actors can perform and impact in a support programme. Therefore, from relational and contextual perspectives, the current study aims to explore the influences of different actors on the content and execution of a bereavement programme in a Swedish context.

### Theoretical framework

In the current study, Pierre Bourdieu’s concepts of power, capital, and position serve as the theoretical framework (42–44). According to Bourdieu (44), the real is relational, which means that social reality consists of power relationships between different objective positions and dispositions, unfolding through dialectic processes. Understanding people means understanding their inherent properties, attributes, or essences in relation to the field of objects, practises, or activities in which they exist (44). Power is central in all social interactions, especially symbolic power. Symbolic power functions as a structure in a social space where related people have accepted what is right or wrong about any phenomenon (things, thoughts, behaviours, traditions, and actions). Symbolic power exists among people in different social contexts, e.g., families, workplaces, and schools (45).

In a social context, actors or groups of actors assume different positions and act differently depending on their access to different valuable capitals, such as economic, social, cultural, and symbolic capital. These capitals are part of the social structure and impact on actors’ behaviours in a specific physical place (42–44, 46).

The physical context of the current study consisted of a physical setting with a specific interior, where different meetings between different actors took place, framing the conditions for their (inter)actions. (43), emphasises that a social context, where concrete human activity takes place, is always a pre-structured context as it is coded with specific rules and values. In the current study, the authors focused on how the structural framework of the support programme—consisting of seven consecutive support sessions with different themes, pre-defined content, and activities—guided and shaped the conditions for possible (inter)actions and vice versa. Hence, the concept of power was employed to illuminate its multidimensional aspects during actors’ (inter)actions in the specific physical setting. Subsequently, the concepts of positions, power, and capitals served as an analytical lens to illuminate how actors’ different social positions and capitals, such as age and roles, might have significance for the conditions of possibility to act in the programme. In the current study, Bourdieu’s relational theoretical framework was used to inspire a focus on symbolic and structural power, capital, and position. Bourdieu’s theoretical concepts highlight the dynamic power relationships between different actors’ positions that influence the possibilities to shape the content and proceedings of the programme, which, in dialectic processes, further influenced the actors’ (inter)actions.

## Materials and methods

### Study design

The current study is a part of a larger ethnographic field study (10, 23), conducted within a Swedish support programme for bereaved children and their families from February to June 2019. Empirical materials were collected through multiple methods, totalling 82 h of observations that led to 130 pages of field notes, 29 semi-structured individual interviews with children and their parents, and one focus group interview with the volunteers. Additionally, short, spontaneous interviews were conducted during mingle sessions and breaks, and these were also documented as field notes. Photographs, drawings, and written notes used during sessions regarding emotions and feelings were also included in the empirical material. Thus, the current study exclusively built on field notes from the observational part of the larger study that is related to the aim of this investigation.

### Structural framework of the programme

The studied support programme, developed and inspired by Swedish psychologist Gyllenswärd (47), was structured based on three key elements: (1) The remaining parents applied through email or phone to join the support programme, and then the remaining parent, their children, and volunteers had a planned initial meeting lasting 30–60 min where they exchanging information about the programme and discussed expectations. (2) Seven sessions were held where children were grouped according to age and parents had a separate group. (3) All seven sessions were structured in terms of time, themes, content, and activities (47). For more details, see Table 1, also presented in another article (23).

**Table 1:** Content of the support programme.

Session	Themes	Content
1	My family and my loss	Introduction about the content in the support programme and introduction of the participants.
2	Grief and grief reactions	Description of and reflection on grief and grief reactions.
3	Grieving and emotions	Description of and reflection on emotions relating to a parent’s death.
4	Relieve and coping strategies	Description of and reflection on relief and coping strategies in bereavement processes.
5	Memories	Reflection on different ways to remember a deceased parent.
6	Support – networks	Description of and reflection on how to identify support and supportive networks.
7	Farewell	Farewell ceremony for the deceased parent and evaluation of the programme.

### Participants and data collection requirements

In total, 10 families participated in the support programme and all of them were informed and invited to participate in the study. Inclusion criteria required that children and their surviving parents participate as families, and the children’s ages ranged between 9 and 18 years. One family declined to participate, and one family was excluded due to inclusion criteria. Eight families, consisting of eight parents (three fathers and five mothers, aged 40–72 years, with an average age of 48) and 11 children (nine boys and two girls, aged 9–14 years), were included in the study. One of the included families only participated in the observational part of the study, while the other seven families participated in all parts of the larger study (10). All eight volunteers (aged 34–69 years, with an average age of 61) participated and had various professional backgrounds such as nursing, priesthood, teaching, deaconry, and leisure education with work experiences ranging from 8 to 44 years. Furthermore, the volunteers had varied experience as facilitators in the programme, spanning from 0 to 15 years. Two of them were beginners and were paired with experienced facilitators during the group sessions (10).

The field study observations were conducted during mingle sessions/breaks and in the seven group sessions for children. The first author conducted the field study with a focus on understanding with whom participants (inter)acted, and how, from the positions they assumed, and in which physical settings these interactions occurred. Furthermore, observations were also conducted during the volunteers’ other activities related to the programme, including initial meetings before the programme started and two planned group meetings on supervisions and reflections, which were led by a social worker specialising in family therapy.

All field observations were conducted in Swedish, and a field journal was written during field work and after all interviews to provide reflexivity support for the field researcher, following the approach of Bourdieu and Wacquant (48). The journal functioned as a reflection tool partly to challenge the field researcher’s preunderstandings but also to guide attention to the researcher’s own emotions in relation to the participants and situations. The journal documented what happened in various situations, what was captured in the observations and why, and how the researcher reacted, both emotionally and mentally. All these reflections supported the preparation and strategies for the next meeting within the studied field.

### Analysis strategy

A latent thematic analysis was conducted, methodically inspired by Braun and Clark (49), and theoretically inspired by Bourdieu’s relational concepts of power, position, and capital (43, 44, 46, 48). Firstly, fieldnotes were read repeatedly by the first author to gain familiarity and get an overall understanding of the empirical material (49), with focus on contextual and relational perspectives regarding actors’ influences that shape the content and proceedings in the studied programme. Secondly, to generate initial codes (49), a table was inspired by Bourdieu’s relational theoretical concepts to carry out the analysis of features in the fieldnotes. The table’s horizontal rows contained information about the support programme’s context (structure, content, place, and environment), the actors’ different positions (volunteers, parents, children, and the researcher), and how power relationships between the actors appeared in the different organised physical spaces. The vertical columns contained information about the actors’ strategies—namely what, when, where, with whom, and how these strategies influenced the content and proceedings of the programme. The table functioned as a multidimensional map, organising initial codes within relational and contextual perspective (43, 44, 46, 48).

Thirdly, the initial coded extracts in the table were sorted in groups to capture both similarities and differences in the empirical material to construct initial sub-themes and themes. Forth, the initial themes were reviewed by all authors in relation to the coded extracts to ensure that the themes were not only cohesive but also distinct from each other, capturing the essence of each theme within the study’s aim (49). Furthermore, the process of defining and naming themes involved thorough discussions among all authors. This ensured a reflective approach (48), which meant that the authors consciously and continuously tried to break with preunderstandings and perceptions during the dialectic analysis processes. Finally, three themes were constructed ‘*Positions defined the premises for confidentiality obligation*’, ‘*Visible and invisible communication patterns in the classroom*’, and ‘*Free time and non-bereavement-related interfaces connected people*’. Moreover, the themes were developed from a relational perspective, focusing on the power relationships within the context of the studied support programme. Additionally, the actors’ different social positions based on cultural capital were considered in each theme. Quotes from the empirical material were used to illustrate transparency in the analysis process. All names have been changed.

### Ethical consideration

The study was approved by the Swedish Regional Ethics Board and conducted in accordance with the ethical guidelines of the World Medical Association (50). Participation was voluntary, and all participants were informed about confidentiality and their right to withdraw at any time without facing any consequences regarding their participation in the support programme. The first author informed all participants orally about the study and provided written information in age-customised versions, with one version for adults and another for children. All participants aged 12 years and above signed a written, informed consent form, and children below12 years gave oral consent. Moreover, children had the opportunity to ask the field researcher questions before and during mingles/breaks regarding their participation in the current study.

## Results

### Positions defined the premises for confidentiality obligation

Six children, two volunteers, and one fieldwork researcher participated in the seven weekly meetings in the support programme. All actors were bound by an obligation to maintain the rule of confidentiality within the programme. The confidentiality obligation functioned as an essential element in the structural framework of the programme, however, the demands of confidentially obligation differed among the three groups of actors: children, volunteers, and the researcher. The volunteers, who had cultural capital as support providers, saw confidentiality obligation as a major part of their role, ensuring that information about the included parents and children was not spread outside the programme. Their confidentiality commitment also functioned as a trust-builder between the volunteers and programme’s participants.

Sam explained the content of the programme to the family and said: ‘*One must participate in the programme to know*, *and we [the volunteers] follow rules of confidentiality*. *No one else will know what you say in the group*’ (Field observation during the initial meeting with the family).

However, the volunteers did not observe confidentiality obligation within the volunteer team and among related professionals, where they could discuss their experiences from the sessions and mingles with each other and an assigned supervisor. During the course, the volunteer team held two legitimate formal meetings with a supervisor, providing support and sharing reflections, emotions, and disillusioned expectations relating to the future strategies of the children, parents, and volunteers.

Children held the position of support recipients in the programme and were expected to refrain from disclosing or discussing any experiences from the programme outside it. In short, this structural premise was a requirement for participating in the programme, where children were supposed to accept the confidentiality obligation. On one hand, the programme offered the children an exclusive safe space to talk and share experiences and feelings. On the other hand, the programme restricted the children by not allowing them to share their experiences and feelings related to the programme outside its physical space. However, it was unknown to the volunteers how the children handled their imposed confidentiality. They neither accepted nor challenged this confidentiality obligation in the face-to-face meetings with the volunteers.

Volunteer 3: ‘*We have some rules that everyone must follow*, *such as what we talk about must stay here and not be shared with anyone outside the group*. *We respect each other and listen to each other*’. The children sat silently during this explanation and did not ask any questions either to each other or to the group leader about the confidentiality obligation (Field observation from the first session).

The fieldwork researcher was positioned as an observer and was the only one with the opportunity to share knowledge outside the programme regarding what had happened, when, where, and in relation to whom. As a private person, the researcher could not share information outside the programme, but as a researcher, the task was to make this support programme visible outside its own framework and space by exploring it. However, the researcher adhered to research ethical laws, including the Helsinki Declaration, ensuring the research participants’ integrity and confidentiality in the research process and subsequent dissemination of knowledge. In that light, the researcher could discuss and document what happened in the programme in anonymised forms, supported by permission obtained through informed consents from all the participants.

The mother seemed positive about the study and turned to her son and said:

The mother: ‘*Everything will be kept confidential*’.

Then the mother looked at me and I confirmed that their personal data will be kept confidential, with only the research team having access to the original data (Field observation initial meeting with the family).

Overall, the volunteers held a position that allowed them to set the conditions for the children’s participation without negotiation. For the children, their role in the programme depended on them accepting these conditions. However, they could follow or break the imposed confidentiality obligation without the other actors’ knowledge. The children were the only actors involved who did not have a formal opportunity to express their experiences and feelings about what happened in the support programme outside of the programme itself. Conversely, the volunteers and the researcher could take their experiences and feelings outside the programme, albeit subject to different limitations and conditions.

### Visible and invisible communication patterns in the classroom

Actions and interactions between children and volunteers were influenced by different organised physical contexts, such as sessions, breaks, and mingles. These different contexts created both space for interactions and clearly defined boundaries for possible interactions. The group sessions took place in a room equipped with educational facilities such as a whiteboard and pens, with a table and chairs arranged by the volunteers in advance. The lead volunteers sat at the head of the table, while the children and field researcher sat at the long sides of the table. This seating arrangement implicitly conveyed a hierarchal positioning with the volunteers symbolically assuming the power of teachers in the classroom. Furthermore, volunteers asserted their symbolic power by presenting their professional backgrounds to the children, positioning themselves as knowledgeable and mediators of information about bereavement. The children held a position as potential recipients of knowledge through the support provided by volunteers during the sessions. In addition, the two volunteers orchestrated the sessions within the hierarchal positions of their roles. The volunteer in a leading role took charge and directed all the activities, while the other volunteer had an assisting role, quietly observing the children’s behaviour, always ready to support the leading volunteer if needed. The field researcher took on the position of observer, obligated to remain silent and not disturb the activities in the sessions. However, the researcher’s position also held symbolic power, granting the right to determine what became research data through field notes, with none of the other actors knowing what was noted down. This symbolic power was reinforced by the presence of an additional adult in the room.

Sam went in first and stood at the head of the white oval table, and then all the children entered the room. At was 17:17 and Sam introduced himself as a nurse. Karina said she also works as a nurse. Sam said that he would talk most and ask questions, and Karina would support if needed, reasoning that it is easier for the children to turn to one person when answering questions or discussing things. The children were quiet while Sam spoke, and they did not ask any questions (Field observation first session).

As a formal social context, the sessions had a fixed opening and closing ritual. The opening ritual was to light an electronic candle in memory of the deceased parent. Children followed this instruction without questioning the significance or purpose of this ritual.

Sam moved on to the next item on the meeting’s agenda, which was lighting candles and as they lit the candles, each participant had to say this line: ‘I’m lighting this candle for my dad/mom and his/her name is…’ (Field observation first group meeting).

The next ritual for those present was to declare their social identity through their names. Facilitated by the volunteers, everyone present in the session, including the field researcher, participated in the name game (sessions 1–3). The aim of the game was to get to know and remember each other’s name. This game created a sense of community, which was an important element in group cooperation, with ‘community’ being a pre-defined concept supporting children in the programme.

Sam started by saying, ‘*My name is Sam*’. Then Sara had to say; ‘Sam, Sara’, and Emma had to say; ‘Sam, Sara, Emma’. Thus, everyone had to repeat the names said before theirs, followed by their own name (Field observation first session).

The closing rituals consisted of two elements: music and extinguishing the candles. The lead volunteer made a secret song list of the children’s favourite songs. Only the lead volunteer knew, which song had been selected by who, and played a selected song from their smartphone at end of each session. The children’s task was to guess whose song it was, which was sometimes successful, sometimes not. The music ritual was mandatory, and some children initially refused to take part in the game. The volunteer used their relational power position and persuaded those children to join in the game. Eventually, all the children had chosen a song. After the music ritual, children extinguished their electric candles before they left the room, symbolising the end of the session.

All the children wrote their songs on a piece of paper except for two. They claimed that they didn’t have a favourite song. Sam continued to ask them to think some more and that they were sure to come up with a song (Field observation first group meeting).

During the sessions, children listened to the volunteers’ instructions, and engaged in individual tasks such as writing or drawing within predefined themes related to their loss, grief, and emotions. The communication had a loss-oriented focus with the objective to support children in reflecting on their emotions, thoughts, and memories of their deceased parent. In the writing tasks, children often wrote single words such as *angry*, *funny*, and *happy* on paper. In the oral explication of the written words/drawings, the volunteers often designated ‘words’ that could point to poor grief processing such as *anger*. In the group setting, the volunteers asked follow-up questions about the children’s written words/drawings, and the children answered briefly, often by saying *yes*, *no*, or *I do not know*. Through the oral exchanges, the volunteers implicitly assessed the children’s psychological condition and suggested explicit solutions for how to cope with the emotions of grief. The children did not enter dialogues with each other during these sessions or ask the volunteers questions. However, they were present, accepting, and did everything they were asked to do during the sessions.

Axel: ‘*On the outside I*’*m happy and funny but on the inside I*’*m angry*’.

Sam: ‘*What are you angry about?*’

Axel: ‘*I don*’*t know*’.

Sam: ‘*Is it worse since your dad died?*’

Axel: ‘*Yes*’.

Sam: ‘*Are you angry at your dad?*’

Axel: ‘*No*’.

Sam: ‘*It*’*s normal to feel the difficult feelings and they must come out*, *and you have to talk about them*’.

All the children sat quietly and listened. (Field observation fourth session).

The volunteers held high ambitions and expectations regarding the active participation and verbal expression of the children in the sessions, viewing their talking as another key element and an indicator of the programme’s success.

Sam: ‘*They*’*re so difficult and don*’*t talk much*. *Take Emma*, *for example*, *when I asked her who has died? She just answered* ‘*dad*’ *quietly and says nothing more*’.

Karina: ‘*Axel barely says two words*’

Sam: ‘*But he looks up more*. *Then he is so pale*. *Maybe scared?*’

Karina: ‘*Oscar wasn*’*t here today*. *He is away*. *He is also quiet*’.

Supervisor: ‘*It is a difficult group regardless*. *Maybe help them pedagogically?*’

Sam: ‘*Today*, *they drew the figure body about their feelings*. *They like to draw*’.

Supervisor: ‘*Can*’*t you talk about their idols or the Melody Festival?*’

*Sam:* ‘*No*, *they don*’*t want to talk*, *we*’*re struggling*, *but no*’ (Group meeting with supervisor).

The volunteers were focused on visible forms of communication during the sessions, such as face-to-face expressions and spoken words. However, some children communicated without using voices. For instance, some covertly used their cell phones under the table, despite an established rule requiring phones to be on silent mode and not used during sessions. Others drew shapes of hearts or stars on paper during the verbal part of the sessions while still seeming to listen. Some communicated their feelings of unease in the session room by keeping their outside clothing on. However, over time, some of those children gradually became more at ease and felt more comfortable, which led them to remove their outside clothing.

‘Volunteer: *You must have your cell phone on silent mode*’ (Field note first session).

At 17:26, Alexander and Oscar were finished with their drawings. Oscar picked up his cell phone, checked it and wrote something on it under the table. Alexander waited a bit, and when he saw that no one had noticed or said anything to Oscar, he did the same, checked his cell phone and read. Then, he smiled at Oscar (Field note third session).

### Free time and non-bereavement related interfaces connected people

In the spacious open lobby on the ground floor, children, parents, and volunteers gathered and mingled together before each session. The lobby functioned as a central gathering place and a waiting area for all actors before the sessions. In contrast to the structured sessions, the lobby was lively with different activities and the sounds of laughter and talking. The conversations between actors covered a range of topics, such as sports, the weather, vacations, and jobs. While mingling with each other, everyone had the opportunity to engage in free and self-selected content in the conversations. However, their positions were not equal as the children were in the company of their parents, placing parents in a higher position than children due to their custodial responsibility, including the upbringing of the children. Moreover, the volunteers had the role of ‘supporters’ and ‘facilitators’, while the others took on the role of ‘potential supported’. However, the ‘support’ agenda was reset during these informal mingles.

Children, parents, and volunteers were gathered in the spacious, modernly furnished lobby between 4:30 and 5.00 pm before the start of each weekly session and again at the end of each session. Eva welcomed Henrik and his mother. Then they went to get snacks and drinks. Then Emma, her mother and little brother came. Emma went straight to Henrik and started talking. They took snacks and drinks and both families sat down at a table. Sara and her father sat by themselves again as they usually do. The younger children were playing with each other. William and two other children sat on benches looking at something on their cell phones (Field observation seventh session).

During the sessions, there were breaks lasting for 10–15 min, providing opportunities for the children to socialise with whomever they wanted. Children and parents had breaks at different times. The volunteers instructed the children: ‘*Go down to lobby*, *get something to eat/drink*, *and come back to the session room*’ (Field notes, first session). While some children followed this instruction, others chose to mingle independently in the lobby or in the corridor outside the session room. These breaks were the only ‘free zone’ away from adults within the programme. The children who interacted with each other had found common ground, i.e., shared interests such as the same taste in music or common affiliations, such as attending the same school.

Alexander and Oscar remained on the ground floor having snacks and drinks and talking to each other and laughing. They didn’t seem to be aware that the break was over, and everyone had gone up. Sam went down to bring them up and noticed that they enjoyed talking to each other (Field observation third meeting).

The volunteers valued the children’s interactions during the breaks. According to the volunteers, ‘*creating communities*’ was interpreted as another success factor of the programme. It also contributed to their personal satisfaction and sense of meaning relating to the effort they had put into the programme. It alleviated their concerns about the limited verbal communication and interactions between the children during the sessions. When hopeful expectations of the children interacting became a visible reality for the volunteers, they demonstrated flexibility by changing the planned time structure and content of the sessions to give children more free time to develop connections and interactions.

Sam: ‘*They* [the children] *are completely engaged in talking to each other and I couldn*’*t disturb them*. *That is the essence—that they should connect with each other*. *I don*’*t think we*’*ll have time for the* ‘*iceberg*’ *theme tonight*. *We*’*ll have to take that next time*. *It*’*s important to make this change*’.

Karina agreed, and the break was extended (Field observation third meeting).

Extended breaks made more children-led (inter)actions possible, and the volunteers paid attention to children who were not engaging with others. In contrast to the sessions, children actively talked with the volunteers about things other than their feelings and emotions. Communication centred around common interests, such as sports and pets. Both the children and volunteers smiled, laughed, and seemed to enjoy the conversations and each other’s company. The ways in which volunteers and children communicated and the topics they discussed changed when the context shifted from the formal sessions to informal breaks, and the formal bereavement agenda was temporarily set aside. The formal sessions on loss, bereavement, and emotions were put on hold, and conversations were based on mutual interests. Over time, informal parts of the programme saw interactions between children and volunteers blossom and friendships develop between the children.

Sam sat down next to Axel who was sitting on a sofa in the hall outside the session room. Sam started talking to Axel about football. Like Axel, Sam likes football, and then I heard Axel talking quite a lot despite usually being quiet during the sessions. Karina and Sara talked about their dogs, and Sara looked happy while Emma was drawing (Field observation third meeting).

In the last session, a balloon ceremony was conducted as a formal conclusion of the programme. This involved the release of balloons with letters to the deceased parent. This could be seen as a rite of passage, symbolising the transition back to their everyday lives after completing the support programme, and implicitly that they should be able to navigate their parental bereavement in new and better ways. A strong connection developed between some of the children during this ceremony when one of the children accidently let go of their balloon before they reached the hill where the balloons were to be released. The child without a balloon was supported by another child who they had developed a friendship with during the programme. The friend assisted the child without a balloon and encouraged them to write a new letter. Afterwards, this smaller group went to the hill where they released their balloons together with the rest of the group.

Alexander accidentally let go of his balloon and it quickly flew up into the sky as it was quite windy. Everyone just said ‘what, oh no’, and he looked so surprised, and the balloon disappeared quickly. Alexander stood by the roadside and wrote another letter against a wall. Oscar waited with him while he wrote it. After the balloon ceremony we came back, and Oscar and Alexander were sitting together at a table talking to each other (Field note seventh and the last session).

Symbolically, children engaged in individual communication with their deceased parent while simultaneously visualising a sense of joint action within the shared community. However, after the last session, all actors separated from each other, marking the formal dissolution of the programme.

## Discussion

The discussion highlights three main findings from the current study. Firstly, the significance of confidentiality obligation emerged as an essential element in the programme’s structure with variations in premises depending on the actors’ positions, symbolic power, and cultural capital. Secondly, the impact of different physical settings, coupled with their related content, influenced, and shaped the conditions of possibility of actors’ (inter)actions. Finally, the third main finding to be discussed was that shared interests, hobbies, or daily encounters, such as attending the same school or living in the same residential area, connected the actors more than the fact that the children had all lost a parent.

The main finding showed the importance of confidentiality obligation in the structure of the programme, functioning on different premises for children, volunteers, and the researcher. Confidentiality obligation is a cornerstone of medical ethics and a fundamental aspect of healthcare services, and it is obligatory for all healthcare professionals to protect the privacy of individuals (51, 52). In the current study, volunteers and the researcher adhered to their formal professional confidentiality obligations to build trust and protect the privacy of participating children and their parents. This aligns with the principles of medical ethics (53). Furthermore, the results showed that the volunteers imposed confidentiality obligation on children, which can be seen as a further way to protect their privacy in a group context. However, this can also be interpreted as an embedded risk of conflicting with the principle of non-maleficence and potentially causing harm (53). Contrary to the programme’s intentions, the imposed confidentially obligation restricted the children from sharing their programme-related experiences and feelings outside of the physical space of the programme. This limitation may add a layer of potential suffering, as studies show that many children have difficulties in finding ways to share and cope with their bereavement (20, 54). Moreover, the confidentiality obligation for children can also be regarded as a challenge in relation to the principles outlined in the United Nations Convention on the Rights of Child (UNCRC) (55) regarding children’s right to freedom of speech, which is applicable in Sweden (56). The UNCRC and Swedish law declare that decisions made by adults must reflect on how their impact will affect children to avoid causing harm (55, 56). Other studies also highlight this problem, showing that professionals often lack the expertise to meet children’s needs and live up to their rights in healthcare, including the context of paediatric care (57, 58). The results also showed that volunteers had the possibility to share information about the content and activities within the formal physical setting of the programme, where the researcher could share this information outside this formal setting. However, children did not have the same opportunity. These hierarchal and positional differences in conditions of possibility can be reflected in the light of Bourdieu and Passeron’s work (59), showing that such inequalities exist in the education system. The studied support programme was designed as a school-like set-up, allowing for reflections on the continuation of the educational system and the mirroring of positional inequalities, particularly regarding the conditions of handling confidentiality obligation.

Furthermore, the main findings showed that (inter)actions of actors were influenced by the different organised physical settings and their related content. The sessions and mingles/breaks had different set-ups that shaped the conditions for the actors’ position-related (inter)actions. Studies show that school breaks, as opposed to classes, are often unstructured, allowing children to make free choices regarding activities and interactions, while adults mainly regard their roles as safety monitors (60, 61). Bourdieu (46) also shows that actors behave differently in social contexts based on their position and access to valuable capital and symbolic power. The current study suggests that physical settings hold significance for patterns of (inter)actions, and one may ponder on the implications this has for the success of the programme. Future studies may explore the significance of sessions, breaks, and mingles in support programmes to understand their implications for success. According to Moos (62), any social context is a powerful setting, and individuals mutually influence each other for better or worse. Therefore, interventions involving people should highlight contextual aspects in the measurements of its impact (63).

The present study also showed that volunteers held expectations that children should express themselves during the sessions, with the children taking on the role as well-behaved pupils who do what they were asked but provided verbal responses that were short and concise. According to Højlund (64), children’s experiences and behaviours are closely tied to the concrete social context or institution they are in. Winter et al. (65) shows that professionals, like the volunteers in the current study, often have an unrealistic perception of and personal preference towards children who strike up meaningful communicative encounters with them. According to Chater et al. (29), support programmes employing talk therapies, both at individual and group levels, are not always welcomed by all children as they can feel discomfort in talking about their feelings. Other studies show that children may prefer structured talk therapy based on verbal communication between the actors (36, 66).

In addition, the main findings show that children had other communication strategies than the adult-led’s strategies such as communication without using their voices, for example through their cell phones or by drawing. According to Højlund (64), children actively contribute to the construction of a social context as they adapt to the social expectations and categorisations imposed upon them, both visibly and invisibly for the adults. In line with the current results, Adebäck (67) shows that bereaved children often hide and do not outwardly express their feelings in their appearance or behaviour in their day-to-day lives. However, the current study highlights that children developed their own strategies within the adult-led programme, both during the sessions and the mingles/breaks. Further research on children’s independent, purposeful, and beneficial(in)visible strategies is needed to more fully understand the complexity of children’s bereavement strategies. Venkatesan (68) argues that there is often a misconception among adults that children lack the ability to handle their emotions regarding grief and bereavement, and they need adults to guide them (68). However, adults need to take into consideration not only what children need to know and understand, but also what and how children want to learn about topics related to death and grief (69). Researchers argue that death, grief, and bereavement should be regarded as natural life events, and suffering should be acknowledged as a part of living life without pathologising it, as individuals often possess the capacity needed to be able to handle their suffering (23, 70, 71).

The main findings also showed that the connections among the actors were forged based on shared interests, hobbies, or regular encounters at shared physical locations such as school or residential areas, rather than solely on the children’s common experience of parental loss. This aligns with LaFreniere and Cain’s (72) study, showing that bereaved children do not want to be marked as different due to their bereavement. Instead, they want to be perceived as ‘normal’ and not distinguished from their peers (72). Bourdieu (73) shows how individuals with similar ways of living, tastes, and preferences tend to connect with each other, and these similarities also function as a means of distinction from those with different tastes, preferences, and ways of living and thinking. The current findings suggest that children find their own ways and strategies within the organised support programme that focuses on bereavement and related contents. In a systematic literature review on children’s everyday lives when a parent is seriously ill with the prospect of imminent death, Author et al. (10, 23) [Blinded for reviewers] also highlight that children in vulnerable situations are still capable of adapting and finding strategies in their daily living. It was obvious that bereavement experiences alone were not enough to connect the children in the studied support programme. This calls for the need to explore how to engage and involve children in future programmes that aim to meet their needs and interests by embracing democratic processes and fostering collaboration with researchers, professionals, and children. Recognising children as proactive collaborators in the creation, development, and execution of different support programmes is crucial (39, 74). It seems important that professionals, researchers, politicians, and decision makers recognise the significance of essential and natural activities such as leisure activities in everyday life, providing coping strategies, and connecting people. This has the potential to improve children’s health and quality of life (75), even in bereavement situations.

Finally, the current study has both strengths and limitations. The ethnographic methodology employed in the research made it possible to gain knowledge through direct encounters in the studied programme, allowing the researcher to observe actions and interactions between the actors in ‘real life’ situations. This multifaceted approach to data collection enhanced the trustworthiness of the results (76). In the observational part of the study, it was only possible to observe the actors’ bodily communication and expressions, while the spontaneous interviews during the field study provided additional insights into the actors’ inner thoughts and emotions, contributing to data triangulation, and strengthening the study’s credibility (76). The study’s transparency in outlining the design process, which entails providing a clear description of the support programme’s context, content, and demographic information of the actors including age and profession, contributes to the transferability and thereby the trustworthiness of the results (77). Moreover, the field researcher’s lack of prior familiarity with the programme and the participants (volunteers, parents, and children) adds to the empirical and analytical distance, enhancing the credibility of the study. The field researcher did have experience in caring for bereaved children, which called for an ongoing awareness and reflection of possible medical/psychological preunderstanding regarding children’s loss and grief. A diary was used for such reflection throughout the research process and functioned as a valuable tool for breaking with the researcher’s preunderstanding. According to Bourdieu and Wacquant (48), a critical reflective approach is essential throughout the research process, involving a double break. The double break means the researchers must break with both the spontaneous experiences of the studied participants and the spontaneous theorising of the researchers. The research team continuously reminded each other about the need for critical self-reflection during the analysis of the empirical material regarding medical pre-understanding and theoretical perspectives.

## Conclusion

Participants in the programme were significantly influenced by the structural framework of the programme, including the rule of confidentiality obligation and different organised physical settings such as sessions, breaks, and mingles. The positions of the actors within the programme provided them with different conditions of possibility of how to ensure the demand of confidentiality obligation. The adults had both formal and informal settings to share their experiences of the programme and what happened, while children were expected not to share their experiences outside the programme. The physical settings and their related content defined the roles and positions of the actors, which influenced and shaped the conditions for their (inter)actions. During the sessions, communication between volunteers and children resembled a teacher-student dynamic, where children followed verbal instructions and briefly answered the teacher. At the same time, the children developed strategies for additional non-verbal communication without adult instruction, which also included interactions with the other children. During breaks and mingles, (inter)actions occurred between some children, leading to the development of friendships based on shared interests or common meeting places such as school or residential area. Other children did not form connections with each other, despite all the children having the shared experiences of a parental death and participating in the support programme. The current results highlight the importance of children’s daily activities and interests as both ways to cope with parental bereavement and connect them to other people. Future research focusing on children’s strategies in their daily lives for coping with the loss of a parent and related adult support strategies is needed. Additionally, exploring the development of support programmes using democratic processes that involve children in designing the concepts and implementation of such programmes would be valuable.

## Data availability statement

The original contributions presented in the study are included in the article/supplementary material, further inquiries can be directed to the corresponding author.

## Ethics statement

The studies involving humans were approved by the Swedish Regional Ethics Board. The studies were conducted in accordance with the local legislation and institutional requirements. Written informed consent for participation in this study was provided by the participants’ legal guardians/next of kin.

## Author contributions

HK: Conceptualization, Data curation, Formal analysis, Funding acquisition, Investigation, Methodology, Project administration, Resources, Validation, Writing – original draft, Writing – review & editing. PL: Formal analysis, Supervision, Writing – original draft, Writing – review & editing. SG: Conceptualization, Formal analysis, Methodology, Supervision, Investigation, Writing – original draft.
